# Effects of caffeine on central and peripheral fatigue following closed- and open-loop cycling exercises

**DOI:** 10.1590/1414-431X2021e11901

**Published:** 2022-02-28

**Authors:** P.G. Couto, M.D. Silva-Cavalcante, B. Mezêncio, R.A. Azevedo, R. Cruz, R. Bertuzzi, A.E. Lima-Silva, M.A.P.D. Kiss

**Affiliations:** 1 Grupo de Estudos em Desempenho Aeróbio da USP, Escola de Educação Física e Esportes, Universidade de São Paulo, São Paulo, SP, Brasil; 2 Faculdade de Nutrição, Universidade Federal de Alagoas, Maceió, AL, Brasil; 3 Laboratório de Biomecânica, Escola de Educação Física e Esportes, Universidade de São Paulo, São Paulo, SP, Brasil; 4 Grupo de Pesquisa em Performance Humana, Universidade Tecnológica Federal do Paraná, Curitiba, PR, Brasil

**Keywords:** Endurance performance, Neuromuscular fatigue, Central fatigue, Peripheral fatigue, Ergogenic aid

## Abstract

We examined whether endurance performance and neuromuscular fatigue would be affected by caffeine ingestion during closed- and open-loop exercises. Nine cyclists performed a closed-loop (4,000-m cycling time trial) and an open-loop exercise (work rate fixed at mean power of the closed-loop trial) 60 min after ingesting caffeine (CAF, 5 mg/kg) or placebo (PLA, cellulose). Central and peripheral fatigue was quantified via pre- to post-exercise decrease in quadriceps voluntary activation and potentiated twitch force, respectively. Test sensitivity for detecting caffeine-induced improvements in exercise performance was calculated as the mean change in time divided by the error of measurement. Caffeine ingestion reduced the time of the closed-loop trial (PLA: 375.1±14.5 s *vs* CAF: 368.2±14.9 s, P=0.024) and increased exercise tolerance during the open-loop trial (PLA: 418.2±99.5 s *vs* CAF: 552.5±106.5 s, P=0.001), with similar calculated sensitivity indices (1.5, 90%CI: 0.7-2.9 *vs* 2.8, 90%CI: 1.9-5.1). The reduction in voluntary activation was more pronounced (P=0.019) in open- (-6.8±8.3%) than in closed-loop exercises (-1.9±4.4%), but there was no difference between open- and closed-loop exercises for the potentiated twitch force reduction (-25.6±12.8 *vs* -26.6±12.0%, P>0.05). Caffeine had no effect on central and peripheral fatigue development in either mode of exercise. In conclusion, caffeine improved endurance performance in both modes of exercise without influence on post-exercise central and peripheral fatigue, with the open-loop exercise imposing a greater challenge to central fatigue tolerance.

## Introduction

The positive effects of caffeine ingestion (∼5 mg/kg of body mass) on exercise performance have been widely investigated (1). Caffeine blocks the adenosine receptors in the central nervous system, enhancing neural drive to active muscles (2). Some evidence also suggests that caffeine might act directly on skeletal muscles, increasing contractile force (3,4). As a result of these central and peripheral effects, caffeine increases performance in a broad range of exercise tasks, including high-intensity whole-body endurance exercise (5).

Surprisingly, even with these multiple effects of caffeine on central and peripheral sites, only a few studies have investigated the consequence of caffeine ingestion on neuromuscular fatigue during a high-intensity whole-body exercise (6-8). Neuromuscular fatigue can be defined as a transitory exercise-induced reduction of the muscle ability to generate power (9), which can be related to failure of the central nervous system to voluntarily activate the muscle (central fatigue) and/or processes distal to or at the neuromuscular junction (peripheral fatigue) leading to an attenuated response of the active muscle to a given neural input (for a review see Weavil and Amann (10)). One study reported that caffeine increased total work in a 10-min cycling time trial (TT), but exercise-induced reduction in evoked quadriceps twitch force (a marker of peripheral fatigue) and voluntary activation (a marker of central fatigue) were similar in magnitude under both caffeine and placebo conditions (6). It is important to highlight that post-exercise neuromuscular fatigue measurements in the mentioned study were assessed 20 min after exercise cessation, when central and peripheral fatigue might have been largely recovered (11). Contrary to these findings, in a study measuring post-exercise fatigue within one minute after exercise cessation, caffeine improved performance during a 4,000-m cycling TT at the expense of a greater end-exercise peripheral fatigue (7).

These aforementioned studies have assessed the effect of caffeine on central and peripheral fatigue after a high-intensity whole-body endurance exercise adopting a ‘closed-loop' model, in which the work rate can be regulated throughout the trial in an attempt to complete the task as quickly as possible (i.e., TT). Another approach to assess endurance performance is by using an ‘open-loop' design, in which the work rate is fixed and exercise is performed until task failure. While it has been reported that an open-loop exercise is as sensitive as a closed-loop exercise for detecting changes in endurance performance induced by a given manipulation (12), a constant-load trial provokes greater physiological strain than a freely paced exercise performed at the same average intensity (13). The mechanism by which a constant-load trial provokes greater physiological strain is not fully known, but it is assumed that an enforced constant-load trial negates the self-managing of the conscious signs of fatigue (13). During closed-loop exercise, however, the individual can fluctuate pace based on subconscious physiological feedback from an array of peripheral receptors (13). Whether this higher physiological strain results in greater central and/or peripheral fatigue after open-loop rather than in closed-loop exercise is unknown. In addition, it has been suggested that caffeine has a significantly greater effect on endurance performance measured during open-loop exercises than during closed-loop exercises (14), but whether caffeine ingestion would result in different end-exercise central and/or peripheral fatigue after open- and closed-loop high-intensity whole-body exercise is also unknown. It would be of interest, therefore, to compare the degree of central and peripheral fatigue after both closed- and open-loop high-intensity whole-body exercise and to determine whether caffeine affects central and peripheral fatigue after both modes of exercises.

The aim of the present study was to compare the degree of central and peripheral fatigue after a high-intensity whole-body endurance exercise adopting closed- (4,000-m cycling TT) and open-loop (task-to-failure trial with work rate fixed at mean power of the 4,000-m cycling TT) exercise modes and whether caffeine ingestion would affect central and peripheral fatigue after both modes of exercises. We also compared the sensitivity of the closed- and open-loop exercises for detecting changes in endurance performance caused by caffeine ingestion. Based on an expectation of higher physiological strain during open-loop exercise (13), our first hypothesis was that central and/or peripheral fatigue would be greater after this mode of exercise compared to closed-loop exercise. As caffeine is much more likely to affect open-loop exercise (14), our second hypothesis was that caffeine might induce greater end-exercise central and/or peripheral fatigue in this mode of exercise than in closed-loop exercise.

## Materials and Methods

### Participants

The required sample size was calculated using the G-Power software (version 3.1.7). With an alpha of 0.05, a desired power of 0.90, and a previously reported effect size for the effect of caffeine on performance during a 4,000-m cycling TT (7) as well as on time to task failure during a high-intensity exercise (15) (in both cases, effect size=1.27), the total sample size necessary to achieve statistical power was estimated to be nine participants. Therefore, nine men with a mean (±SD) age of 32.3±6.0 years, body mass of 79.3±6.8 kg, height of 181.2±7.9 cm, peak power of 394±44 W (5.0±0.3 W/kg), respiratory compensation point (RCP) of 280±34 W (3.5±0.3 W/kg and 71.2±5.6% peak power), maximal oxygen uptake of 4.3±0.7 L/min (55.2±5.7 mL·kg^-1^·min^-1^), and habitual caffeine consumption of 85.5±71.3 mg/day were recruited to participate in this study. Participants had ∼4.5 years of cycling experience, with approximately 300 km of training per week, and were classified as trained cyclists in accordance with De Pauw et al. (16). The study was approved by the Ethics Committee for Human Studies of the University of São Paulo (#807.005). A written informed consent form was signed by each participant before the beginning of the study.

### Experimental protocol

Participants visited the laboratory nine times at least 48 h apart, within a 4-week period. In the first visit, the participant's health status was evaluated via a medical screening and a resting electrocardiogram, followed by anthropometric measurements. Then, participants performed a maximal incremental exercise test on their own bikes attached to a CompuTrainer (RacerMate4^®^, CompuTrainer™, USA) to determine their maximal oxygen uptake, maximal power output, and RCP. The maximal incremental exercise test started with a 5-min warm up at 100 W, followed by increments of 30 W every minute until task failure. Participants were instructed to maintain pedal rotation between 80 and 90 rpm, with task failure defined as a drop in pedal rotation below 80 rpm for more than 5 s, despite verbal encouragement (7).

On visits 2 and 3, participants were familiarized with the 4,000-m cycling TT and with neuromuscular function assessment. On visits 4 and 5, using a crossover, double-blind design, participants performed a 4,000-m cycling TT one hour after ingestion of placebo (capsule containing cellulose) or caffeine (capsule containing 5 mg/kg body mass of caffeine anhydrous). The CompuTrainer was set at a cadence-dependent mode and participants were free to shift gear ratio and pedal frequency during the trials. Constant feedback of covered distance was available on a computer screen positioned in front of the participants, but no other feedback, such as power, speed, or heart rate, was provided. Neuromuscular function was assessed before supplementation (Baseline), 60 min after the capsule ingestion (Pre-exercise), and 2 min after the end of the exercise (Post-exercise).

On visits 6 and 7, participants were familiarized with the task-to-failure trial. On visits 8 and 9, participants performed the task-to-failure trial one hour after ingestion of placebo or caffeine. The external work rate was fixed by setting the CompuTrainer in a cadence-independent mode, in which the selected target work rate is maintained constant throughout the test. Mean power and pedal cadence, measured from the 4,000-m TT of visits 2 and 3, were used to set the work rate and pedal frequency (313±41 W, 79±4% of peak power, 100±10 rpm). The gear ratio was also fixed during the entire trial (i.e., 50×14). Task failure was defined as a drop in pedal rotation below 90% of individual target cadence for more than 5 s, despite verbal encouragement (17). The neuromuscular function was assessed as in TT (i.e., Baseline and Pre- and Post-exercise).

Before all trials, the rear tire pressure was set at 110 psi and the CompuTrainer was calibrated according to manufacturer's instructions. Briefly, the rolling resistance applied to the bicycle tire (1.96-2.0 lbs) was determined by a calibration acceleration process performed before and after a 10-min warm-up at 150 W. The calibration acceleration consisted of an acceleration of the system up to a speed of 25 mile/h immediately followed by free-wheels for a standard calibration figure to be registered. All calibration procedures were done by the same researcher. When the recommended calibration procedures are followed, the CompuTrainer presents an error of measurement in power output inferior to 1% (18).

After the calibration procedures, participants performed a 5-min warm-up at 150 W maintaining 90 rpm. Participants were instructed to remain seated throughout the trials. The trials were performed at the same time of the day, and participants were instructed to abstain from caffeine, alcohol, and strenuous physical exercise 24 h before each trial. They were also asked to follow the same diet during the 24 h before each trial and to have their last meal at least two hours before the trials. Compliance with these pretest instructions was checked by having participants fill out pre-test diet and exercise records. Participants were asked after each exercise trial about which substance they thought they had ingested.

### Neuromuscular function assessment

A Neuro-TES electric stimulator (Neurosoft, Russia) was used to stimulate the femoral nerve and assess neuromuscular function of the right quadriceps muscles, as described in previous studies from our laboratory (7,19,20). Briefly, participants were seated with the hip at 120° and the knee at 90° on a modified knee-extension chair (Cefise, Brazil). The lever arm of the machine was fixed to a force transducer (SML-500, Interface, USA) and the right ankle attached to the lever arm by a non-compliant cuff. Inelastic straps were used to hold the participants on the chair. A cathode electrode was placed on the femoral triangle and an anode electrode on the gluteal fold. The optimal electrical stimulation intensity for further use in the experimental trials was determined by single pulse (1 Hz, 80 µs of duration) delivery to the femoral nerve, starting at 100 V and progressively increasing 30 V every 30 s until attainment of a plateau in evoked twitch quadriceps force (Q_tw_) and muscle action potential (M-wave) amplitude of *vastus lateralis*. The electromyography activity of the right *vastus lateralis* muscle was monitored by a bipolar Ag-AgCl surface electrode (Hal, Brazil) with a sample rate of 1 kHz (MyoTraceTM 400, Noaraxon, USA). To determine M-wave amplitude, peak-to-peak amplitude of the electromyography signal induced by the electrical stimulation was quantified.

To ensure maximal stimulation in the experimental trials, the stimulation intensity was set at 120% of the plateau in Q_tw_ and M-wave. The plateau in Q_tw_ and M-wave was double-checked at the beginning of every trial session. Before baseline assessments, a warm-up was performed (5-s isometric contractions at 50, 60, 70, 80, and 100% of maximal voluntary contraction, with a 30-s interval between contractions), and then six 5-s maximal voluntary contractions (MVC) were performed, with visual feedback of force provided on a computer screen positioned in front of the participant. Participants were asked to reach their maximum force rapidly and maintain it for 5 s, with verbal encouragement provided during all contractions. Electrical stimulus (1 Hz, 80 µs of duration) was applied on the femoral nerve when the isometric force reached a plateau (superimposed twitch) and 2 s after the end of MVC in relaxed muscle (potentiated quadriceps twitch force, Q_tw,pot_). During baseline and pre-exercise phases, the first two measurements were discarded to avoid the effects of potentiation on Q_tw,pot_; thus, the average of the remaining four measurements was used for further analysis (21). A single MVC with electrical stimulation was performed 2-min post-exercise (19) and later used to quantify exercise-induced neuromuscular fatigue (see below).

The MVC was recorded as the highest value found during each contraction (22). The Q_tw,pot_ was recorded as the evoked peak force (23). The voluntary activation (VA) was calculated using a modified version of the superimposed twitch equation (24):

$$\text{VA}(\%)=100–\left[\text{D}\times \left(\text{FIB}/\text{MVC}\right)/{\text{Q}}_{\text{twpot}}\right]\times 100$$ (Eq. 1)

where, FIB is voluntary force immediately before superimposed twitch, D is the force difference between FIB and maximum force evoked by the superimposed twitch, and MVC is the maximal voluntary contraction.

Between-day, within-subject coefficients of variation in our laboratory were ∼5% for MVC, ∼2% for VA, and ∼5% for Q_tw,pot_ (7,20).

### Physiological strain

Exercise-induced physiological strain was determined by measuring total mechanical work, heart rate, oxygen uptake, and pulmonary ventilation responses during the trials. Total mechanical work was calculated by multiplying mean power by exercise time. Heart rate was continually recorded using a heart rate monitor (Polar FT1 Coded, Finland), while oxygen uptake and pulmonary ventilation were measured breath-by-breath using a pre-calibrated metabolic cart (Cortex Metalyzer 3B, Cortex Biophysik, Germany). Values of heart rate, oxygen uptake, and pulmonary ventilation recorded in each trial were averaged for further analysis.

### Statistical analysis

Normal distribution of the data was confirmed using the Shapiro-Wilk test. Performance during closed- and open-loop exercises was compared between caffeine and placebo using the paired *t*-test. Hedges' g effect size (ES) and 95% confidence interval (95%CI) were calculated using an online calculator (https://effect-size-calculator.herokuapp.com/) from means and pooled standard deviations to verify the magnitude of the effect of caffeine on performance during closed- and open-loop exercises, assuming values of 0.2, 0.6, 1.2, 2.0, 4.0, and >4.0 as trivial, small, moderate, large, very large, and extremely large, respectively (25).

The degree of sensitivity of the closed- and open-loop exercises for detecting changes in endurance performance with caffeine ingestion was determined by calculating a sensitivity index, as previously recommended (26). Briefly, performance times were log-transformed, and the sensitivity index was calculated by dividing mean differences between placebo and caffeine by the error of measurement. The error of measurement of a given trial (closed- or open-loop trial) was calculated by dividing the standard deviation of the differences between the two familiarization trials by √n-1 (26). The sensitivity index was further corrected downwards for small-sample bias using the following equation (27):

$$1\surd \text{n}\times \left\{1-3/\left[4\times \left(\text{n}-1\right)\right]\right\}\times \surd 2$$ (Eq. 2)

The confidence limits of the sensitivity index were derived using a macro to generate quantiles in an Excel spreadsheet. Comparison of the sensitivity index for the two modes of exercise was made by inspecting the overlap of the closed- *vs* open-loop confidence intervals, as previously described (12).

To check the existence of a potential order effect on exercise performance, time to cover the 4,000-m cycling TT and time to task failure during the first and second trials were compared using a paired *t*-test. As no control trial without supplementation was inserted in the experimental design (28), a possible placebo effect was checked by comparing time to cover the 4,000-m cycling TT and time to task failure during the second familiarization with their corresponding placebo trials using a paired *t*-test. The blinding effectiveness was tested using the χ^2^ test.

As the preliminary analysis with the paired *t*-test showed that oral supplementation alone had no effect on neuromuscular function (i.e., baseline *vs* pre-exercise), which is similar to the results of previous studies (7), three-way within-subject repeated-measure ANOVA was further used to determine the effect of supplement (caffeine *vs* placebo), trial (closed- *vs* open-loop), and time (baseline *vs* post-exercise) on MVC, VA, and Q_tw,pot_. If ANOVA yielded a significant result, follow-up pair-wise comparisons were conducted using the Bonferroni correction. Analyses were performed using SigmaStat 3.5 (Systat Software, Inc., USA). All data are reported as means±SD, and statistical significance was set at P<0.05.

## Results

### Reliability of exercise performance, order effect, and blinding effectiveness

The typical error of measurement was 4.0 s (90%CI: 2.9-6.8) for time to covering the 4,000-m cycling TT (coefficient of variation=0.9±0.5%). The corresponding values for time-to-task failure trial were 39.6 s (90%CI: 28.5-67.8, coefficient of variation=9.4±4.1%). There was no significant order effect for both the 4,000-m cycling TT (trial 1: 371.6±16.2 s; trial 2: 371.8±14.2; P=0.965) and the time-to-task failure trial (trial 1: 493.9±133.6 s; trial 2: 476.9±115.8 s; P=0.763). In addition, time to cover the 4,000-m cycling TT during the second familiarization session (372.7±15.6 s) was not significantly different from the placebo trial (371.6±16.2 s), and time to task failure during the second familiarization session (453.2±127.7 s) was not significantly different from the placebo trial (418.2±99.6 s). The percent of correct identifications of which supplement was ingested was not different from that expected due to chance in both the 4,000-m cycling TT (χ^2^=0.22, P=0.637) and the time-to-task failure trial (χ^2^=2.80, P=0.089).

### Overall performance

**Figure 1:**
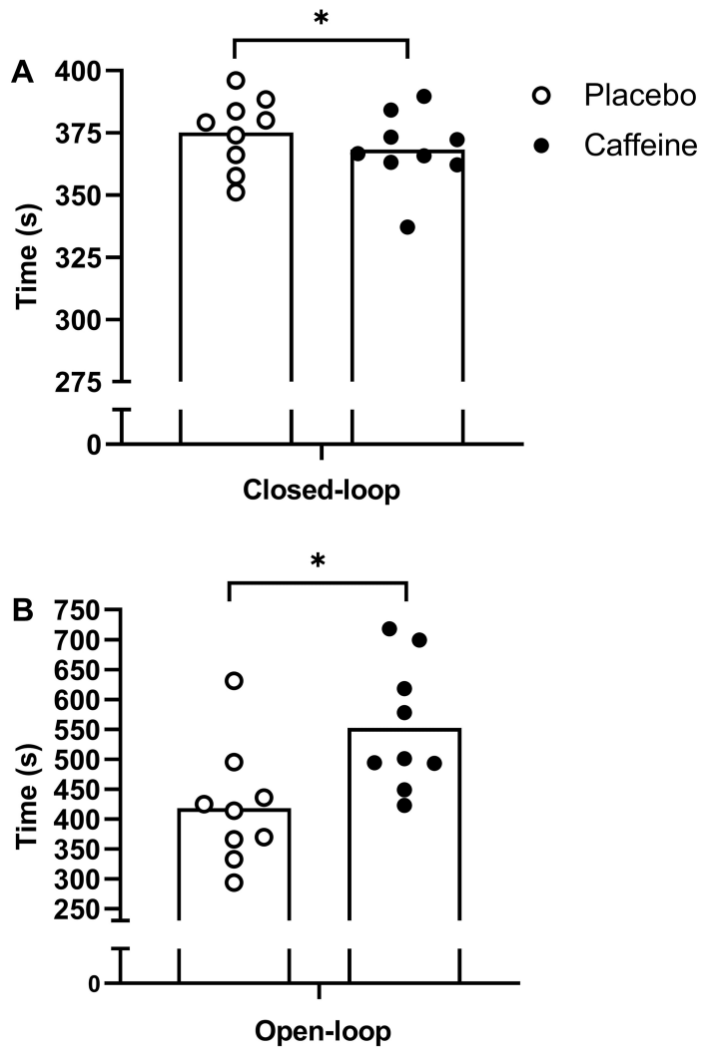
Time to complete 4,000 m cycling time trial (closed-loop exercise) (**A**) and time to task failure (open-loop exercise) (**B**). Data are reported as means±SD. *P<0.05 (paired *t*-test).

Mean power during the 4,000-m cycling TT in the caffeine condition (323±40 W, 115.4±14% RCP) was higher (P=0.029) than in the placebo condition (308±37 W, 110.3±13% RCP). Time to cover the 4,000-m cycling TT (Figure 1A) was significantly faster under the caffeine condition compared to the placebo condition (368.3±15.0 and 375.1±14.5 s, ES=0.42, 90%CI: 0.12-0.78, P=0.024). Time to task failure (Figure 1B) under the caffeine condition took longer than the placebo condition (552.6±106.6 and 418.2±99.6 s, ES=1.18, 90%CI: 0.59-1.96, P=0.001). The sensitivity index for detecting placebo to caffeine changes was similar between closed- (1.5, 90%CI: 0.7-2.9) and open-loop exercises (2.8, 90%CI: 1.9-5.1).

### Neuromuscular fatigue

**Figure 2:**
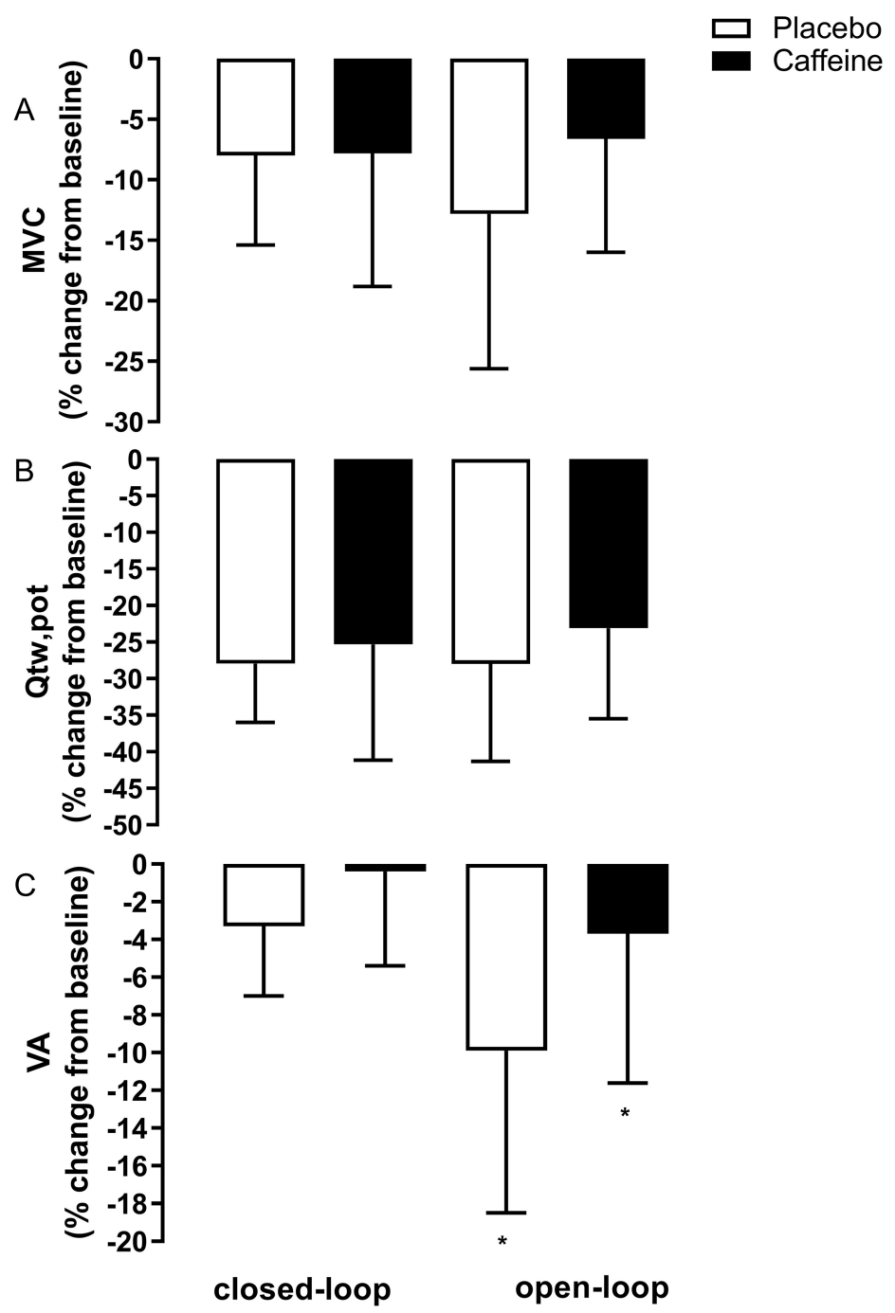
Reduction (means±SD of percentage change from baseline to post-trial) in (**A**) maximal voluntary contraction (MVC), (**B**) evoked quadriceps twitch force (Q_tw,pot_), and (**C**) voluntary activation (VA) after a 4,000-m cycling time trial (closed-loop exercise) and a task-to-failure trial (open-loop exercise) with caffeine and placebo ingestion. Trial *vs* time interaction, with a greater reduction from baseline to post-exercise in the open- than in the closed-loop exercise. Data are reported as means±SD. *P<0.05 (three-way repeated-measure ANOVA).

Data of neuromuscular fatigue are shown in Table 1 and Figure 2. There were no interactions between factors (P≥0.05) or main effect of trial (P=0.679) or supplement (P=0.137) for MVC. There was only a main effect of time for MVC (P=0.001), with a reduction from baseline to post-exercise for all trials and supplements. Similar results were obtained for Q_tw,pot_, with no interactions (P≥0.05) or main effect of trial (P=0.552) or supplement (P=0.097). There was only a main effect of time for Q_tw,pot_ (P=0.001), with a reduction from baseline to post-exercise for all trials and supplements. There was a trial *vs* time interaction for VA (P=0.019), with the open-loop exercise showing greater exercise-induced reduction than the closed-loop exercise. There was no main effect of supplement (P=0.307) or any other interactions (P≥0.05) for VA.

**Table 1:** Neuromuscular function before (baseline) and after a 4,000-m cycling time trial (closed-loop exercise) and a time-to-task failure trial (open-loop exercise) with caffeine (CAF) and placebo (PLA) ingestion.

	Closed-loop exercise		Open-loop exercise	
	PLA	CAF	PLA	CAF
MVC (N)*				
Baseline	678.0±134.3	687.5±141.9	677.5±157.4	681.7±151.8
Post-exercise	624.0±144.4	640.4±183.6	594.0±172.4	631.0±123.2
Q_tw,pot_ (N)*				
Baseline	187.2±31.5	181.8±23.9	181.9±28.9	179.1±26.2
Post-exercise	135.5±30.0	137.7±41.6	132.0±36.6	138.6±33.8
VA (%)^#^				
Baseline	92.5±2.7	92.6±2.7	91.7±4.2	89.7±5.9
Post-exercise	89.5±5.2	92.2±3.5	82.7±9.4	86.1±7.2

Data are reported as means±SD. *Main effect of time (lower values post-trial compared to baseline, P<0.05). ^#^Trial *vs* time interaction (greater reduction from baseline to post-trial in the open- than in the closed-loop exercise, P<0.05). Three-way within-subject repeated-measure ANOVA. N: Newtons; MVC: maximal voluntary contraction; Q_tw,pot_: potentiated quadriceps twitch force; VA: voluntary activation.

### Physiological strain

Data of physiological strain are shown in Table 2. There was a main effect of trial (P=0.001) and trial *vs* supplement interaction (P=0.002) for total work, with higher values in open- compared to closed-loop exercise (P=0.001), and under the caffeine condition during the open-loop exercise (P=0.005), but not during the closed-loop exercise (P=0.139). There was a main effect of trial for heart rate (P=0.047), with higher values in the open-loop exercise. There was also a main effect of supplement for heart rate (P=0.020), oxygen uptake (P=0.008), and pulmonary ventilation (P=0.008), with higher values under the caffeine condition compared to the placebo condition.

**Table 2:** Total work and physiological responses during a 4,000-m cycling time trial (closed-loop exercise) and task-to-failure trial (open-loop exercise) with caffeine (CAF) and placebo (PLA) ingestion.

	Closed-loop exercise		Open-loop exercise	
	PLA	CAF	PLA	CAF
Total work (kJ)^†^	120.6±9.9	124.6±10.4	149.7±26.6	191.3±20.9
Heart rate (bpm)*^††^	163±13	164±9	163±10	168±9
V̇O_2_ (L/min)^††^	3.95±0.42	4.04±0.64	3.85±0.77	4.09±0.51
V̇E (L/min)^††^	136.5±16.2	144.6±15.1	134.2±17.5	144.0±9.1

Data are reported as means±SD. *Main effect of trial (higher values in the open-loop rather than in the closed-loop exercise, P<0.05). ^†^Trial *vs* supplement interaction (higher values in the caffeine than in the placebo condition only for open-loop exercise, P<0.05). ^††^Main effect of supplement (higher values in the caffeine than in the placebo condition, P<0.05). ANOVA. V̇O_2_: oxygen uptake; V̇E: pulmonary ventilation.

## Discussion

The present study indicated that, for detecting caffeine-induced improvements in endurance performance, the sensitivity of a closed-loop exercise is similar to that of an open-loop exercise. However, the open-loop exercise induced greater post-exercise central fatigue. Nevertheless, caffeine ingestion did not affect end-exercise central or peripheral fatigue in either the closed- or open-loop model.

Caffeine ingestion improved performance during the 4,000-m cycling TT (∼1.8%), similar to what has been previously reported for TT of similar distance and duration (6,29). Caffeine also increased time to task failure during a constant-load trial (∼35%), a result that also corroborates previous findings (15,30). While changes in time to task failure resulting from experimental interventions are expected to be greater than in TT performance (12), the sensitivity for detecting performance changes seems to be similar when the differences in error of measurement between modes of exercise are taken into account (12,25). Our data suggested, therefore, that caffeine was ergogenic in different models of exercise, and that closed- and open-loop exercises had similar sensitivity in detecting ergogenic effects of caffeine. This is in accordance with a previous report that demonstrated that both models of exercise have comparable sensitivity for detecting changes in endurance performance induced by hypoxia and hyperoxia (12). As previously suggested (12), our data reinforced that the choice between closed- and open-loop exercises should be based on other considerations rather than sensitivity.

Even though both models of exercise showed similar sensitivity, total work done and mean heart rate were greater in the open-loop exercise, suggesting an increased physiological strain. As a result, exercise-induced reduction in VA was more pronounced after the open-loop exercise. Previous studies have suggested that during a 4,000-m cycling TT performed above critical power (i.e., within the severe-intensity domain) or a task-to-failure trial performed at power of maximal oxygen uptake (also within the severe-intensity domain), fatigue is predominantly of peripheral origin (22,31,32). In the present study, mean power during the 4,000-m cycling TT for both placebo and caffeine trials was above the power corresponding to the RCP (a surrogate of critical power), suggesting that the 4,000-m cycling TT was performed within the severe-intensity domain in both placebo and caffeine conditions. Consequently, a significant amount of peripheral fatigue was identified after both placebo and caffeine trials. It is, however, not uncommon to report some degree of central fatigue after an exercise of this intensity (7,8,19,31). Although no previous study has compared exercise-induced reduction in VA between closed- and open-loop exercises, central fatigue seems to vary in an intensity-dependent manner (31). A small reduction in VA has been reported for exercise performed at power of maximal oxygen uptake (31). A greater reduction in VA, however, is found when the exercise is performed at the RCP, an exercise intensity where time to task failure is longer than when exercise is performed at power of maximal oxygen uptake (31). Thus, the magnitude of central fatigue may rise as exercise duration increases. As exercise time for the open-loop was ∼40% longer than for the closed-loop exercise, this longer exercise time may have induced the greater reduction in VA after the open-loop exercise.

Although reduction in VA was more pronounced after the open-loop exercise, VA was not influenced by caffeine in either exercise model. Previous studies using a 4,000-m cycling TT showed no effect of caffeine on exercise-induced reduction in VA (7,33). To our knowledge, there is no data of VA reduction after an open-loop exercise after caffeine ingestion. Nevertheless, a study using a single-leg, intermittent isometric knee extension contractions performed until task failure found that caffeine ingestion increased time to task failure and attenuated the rate of decline in VA throughout the exercise without changes in the VA at task failure (34). Although the same may have occurred in our study, we were unable to measure the rate by which VA declined during exercise. Thus, further studies measuring VA throughout an open-loop, whole-body exercise after caffeine ingestion are necessary to test this hypothesis.

Different from VA, the degree of decline in evoked twitch quadriceps force was similar for both exercise models. These findings are in accordance with the ‘peripheral fatigue threshold concept' (35). A peripheral fatigue threshold has been proposed to represent the maximal level of peripheral fatigue attainable after an exercise (35). It is assumed that the maximal level of end-exercise peripheral fatigue is a fixed amount for a given individual (35). It should be noted, however, that the peripheral fatigue threshold is undoubtedly task-specific, as a greater degree of end-exercise peripheral fatigue is attained after isometric single-joint exercise than after whole-body exercise (10). In the present study, although closed- and open-loop exercises differ in relation to their mode of execution (self-paced *vs* fixed work rate), each is a whole-body exercise presumably recruiting the same amount of muscle mass. Thus, our finding suggests that the peripheral fatigue threshold concept is preserved during different models of whole-body, high-intensity exercise.

There was also no effect of the supplement on the level of decline in evoked twitch quadriceps force. It was hypothesized that caffeine would affect the degree of end-exercise decline in quadriceps twitch force, based on a previous study showing that caffeine ingestion increases performance during a 4,000-m cycling TT at the expense of greater end-exercise locomotor muscle fatigue (7). A more recent study has demonstrated, however, that the caffeine-induced improvement on 4,000-m cycling TT performance seems to be at the expense of greater locomotor muscle fatigue in low- but not in high-performing cyclists (33). In fact, our cyclists performed the 4,000 cycling TT closer to the high-performing (∼370 s) than to the low-performing (∼412 s) cyclists in the aforementioned study. This supports the assumption that caffeine ingestion can improve performance in physically fit cyclists without negatively affecting their end-exercise peripheral fatigue. In relation to the open-loop exercise, no previous study has investigated the effect of caffeine ingestion on decline in evoked twitch force after this mode of exercise. As reported for VA, one study noted that caffeine expanded time to task failure without altering the end-exercise quadriceps twitch torque during a single-leg, intermittent isometric knee extension contraction (34). Our findings add that the peripheral fatigue threshold was not exceeded with caffeine ingestion during a whole-body exercise performed in a closed- or an open-loop cycling exercise model, at least when the peripheral fatigue threshold is measured in cyclists with higher physical fitness such as those recruited in the present study.

Together, our findings indicate that endurance performance - measured as mean power during a closed-loop exercise or time to task failure in an open-loop exercise - improves with caffeine ingestion. This improvement was not accompanied by changes in the amount of end-exercise central or peripheral fatigue. However, the fact that the same degree of end-exercise central and peripheral fatigue was attained in placebo and caffeine conditions even with caffeine condition presenting higher power (closed-loop exercise) or duration (open-loop exercise) suggests that caffeine might have reduced the rate of central and peripheral fatigue development. During closed-loop exercise, a lower rate of central and peripheral fatigue development might have enabled participants to employ a higher power during the trial. During the open-loop exercise, a lower rate of central and peripheral fatigue development might have enabled participants to sustain exercise longer. Thus, the higher power/duration with caffeine ingestion might have compensated the lower rate of central and peripheral fatigue development induced by caffeine ingestion, which resulted in similar end-exercise central and peripheral fatigue between placebo and caffeine conditions. Nevertheless, as we have not measured the rate of decline in central and peripheral fatigue, further studies are necessary to confirm this assumption.

A limitation of the present study is that there was a natural delay when moving from the cycle ergometer to the knee extension chair, which might result in partial recovery of both central and peripheral fatigue (36,37). Although some fatigue recovery might occur within this time, the magnitude of recovery might have been small and similar between conditions, as the transition time was maintained constant across the conditions (i.e., 2 min). This transition time is also similar to several studies investigating central and peripheral fatigue after whole-body exercise (19,22,33,35). In addition, the lack of a control condition precludes the verification of a potential placebo effect (28). Nevertheless, we noted no difference in exercise performance between the second familiarization session (without pill ingestion) and placebo trial (with inert pill ingestion) for both closed- and open-loop exercises, which suggests that a potential placebo effect impacting our results is unlikely. Additionally, the number of correct identifications of the ingested supplement (caffeine or placebo) was not different from that expected by chance, suggesting a successful blinding process. Another factor that could influence the ergogenic effect of caffeine was that participants were low-to-moderate caffeine consumers and there was a 24-h caffeine withdrawal before the trials. However, some evidence suggests that habitual caffeine consumption (38) as well as the presence or absence of a withdrawal period (39) do not affect the ergogenicity of caffeine. Finally, our study was conducted using high-intensity exercise trials performed in the severe-intensity exercise domain. Whether these results can be expanded to other exercise should be further investigated.

In conclusion, caffeine ingestion improved endurance performance, regardless of whether the endurance task was performed with a closed- or an open-loop exercise model. Caffeine-induced improvements in endurance performance did not come at the expense of greater central and peripheral fatigue in either exercise model. However, the open-loop exercise resulted in a greater end-exercise central fatigue than the closed-loop exercise.
